# Identification of the genetic characteristics of copy number variations in experimental specific pathogen-free ducks using whole-genome resequencing

**DOI:** 10.1186/s12864-023-09928-8

**Published:** 2024-01-02

**Authors:** Lanlan Li, Jinqiang Quan, Hongyi Liu, Haibo Yu, Hongyan Chen, Changyou Xia, Shengguo Zhao, Caixia Gao

**Affiliations:** 1 College of Animal Science & Technology, Gansu Agricultural University, Lanzhou, 730070 P.R. China; 2 State Key Laboratory of Veterinary Biotechnology, Heilongjiang Provincial Key Laboratory of Laboratory Animal and Comparative Medicine, National Poultry Laboratory Animal Resource Center, Harbin Veterinary Research Institute, Chinese Academy of Agricultural Sciences (CAAS), Harbin, 150069 P.R. China; 3 College of Animal Science & Technology, Ningxia University, Yinchuan, 750021 P.R. China

**Keywords:** Laboratory ducks, Whole-genome resequencing, Genetic characteristics, Selection signature, Copy number variation

## Abstract

**Background:**

Specific pathogen-free ducks are a valuable laboratory resource for waterfowl disease research and poultry vaccine development. High throughput sequencing allows the systematic identification of structural variants in genomes. Copy number variation (CNV) can explain the variation of important duck genetic traits. Herein, the genome-wide CNVs of the three experimental duck species in China (Jinding ducks (JD), Shaoxing ducks (SX), and Fujian Shanma ducks (SM)) were characterized using resequencing to determine their genetic characteristics and selection signatures.

**Results:**

We obtained 4,810 CNV regions (CNVRs) by merging 73,012 CNVs, covering 4.2% of the duck genome. Functional analysis revealed that the shared CNVR-harbored genes were significantly enriched for 31 gene ontology terms and 16 Kyoto Encyclopedia of Genes and Genomes pathways (e.g., olfactory transduction and immune system). Based on the genome-wide fixation index for each CNVR, growth (*SPAG17* and *PTH1R*), disease resistance (*CATHL3* and *DMBT1*), and thermoregulation (*TRPC4* and *SLIT3*) candidate genes were identified in strongly selected signatures specific to JD, SM, and SX, respectively.

**Conclusions:**

In conclusion, we investigated the genome-wide distribution of experimental duck CNVs, providing a reference to establish the genetic basis of different phenotypic traits, thus contributing to the management of experimental animal genetic resources.

**Supplementary Information:**

The online version contains supplementary material available at 10.1186/s12864-023-09928-8.

## Background

Genetic variation is an important genetic basis for individual differences. Copy number variation (CNV) is a type of genetic variation that varies from 50 kb to a few Mb in size compared with the reference genome sequence of an organism, particularly a deletion event or duplication type affecting many base pairs [1]. Neighboring CNV areas with overlapping regions can be merged into one large genomic segment referred as a copy number variant region (CNVR). CNV is a complementary genetic variant to a single nucleotide polymorphism (SNP), which has a more substantial impact on gene expression and function. For example, changing the genetic structure and dose, destroying a coding sequence, interfering with long-term gene regulation, and exposure of recessive genes, all of which have important implications for animal phenotypic polymorphism, disease susceptibility, and evolutionary adaptation [2–4].

With the development of large-scale human CNV research, substantial progress has been made in CNV detection in livestock and poultry species, including cattle (*Bos taurus*) [5, 6], pigs (*Sus scrofa*) [7], goats (*Capra hircus*) [8], sheep (*Ovis aries*) [9], dogs (*Canis familaris*) [10], and chickens (*Gallus gallus*) [4]. To date, CNV overlapping genes screened in numerous animal models have been shown to be related to coat color [10, 11], meat quality [12], reproduction [13], immune response [14], disease [15], and environmental adaptations [16]. Moreover, CNVs provide resources towards the creation of new genes [17]. Compared with SNP chips and array comparative genomic hybridization (aCGH) microarrays chips, whole genome resequencing (WGRS) technology is more comprehensive and accurate for genome level recognition of CNVs, thereby improving the accuracy of functional genetic prediction [18].

Duck (*Anas platyrhynchos*) is the most widespread and agriculturally important waterfowl species in the world, providing significant economic benefits from its use as a high-quality source of meat, eggs, and feathers [19]. Furthermore, ducks are hosts for most avian diseases and have been shown to undergo high morbidity, long-distance transmission and carry multiple viruses [20, 21]. Consequently, Specific Pathogen Free (SPF) ducks, which have been artificially bred to carry controlled microorganisms and have a clear genetic background, are important experimental materials for avian pathology research and the production of avian-derived biological products [22, 23]. Characterization of whole-genome sequence variation in SPF ducks and the identification of phenotype-related functional variants are crucial to assess their genetic quality and are necessary to guide future genome-assisted breeding and disease studies.

With the availability of duck reference genome sequences, studies have successfully applied SNP loci identified by WGRS technology in population structure analysis [24], trait localization [25], and population evolution [19] of ducks. However, CNVs in the duck genome have not been thoroughly studied on a genome wide basis. After Skinner et al. [26] obtained the first genomic CNV map of ducks using the aCGH detection method, only Xu et al. [27] has explored the CNVs associated with the number of cervical vertebrae in Pekin ducks using genome-wide association analysis. Herein, the CNVs of three representative experimental ducks, Jinding duck (JD), Shaoxing duck (SX), and Fujian Shanma duck (SM), were analyzed using the WGRS technique for the first time. They are often used as ancestral generations to breed new varieties because of their excellent fecundity and adaptive performance. Besides, the successful breeding of JD, SX and SM populations has led to their wide use in research related to avian pathogens, such as Newcastle disease virus [28], avian influenza virus [29], and avian reovirus [30]. Our main objectives were to characterize the genome wide CNV variation within and among populations, and identify CNVs and related functional genes associated with different phenotypic traits in each experimental duck. A significant number of experimental duck CNVs and candidate CNVRs were identified, which will provide a valuable resource for the genetic characterization of different experimental duck populations.

## Materials and methods

### Sample collection

**Fig. 1:**
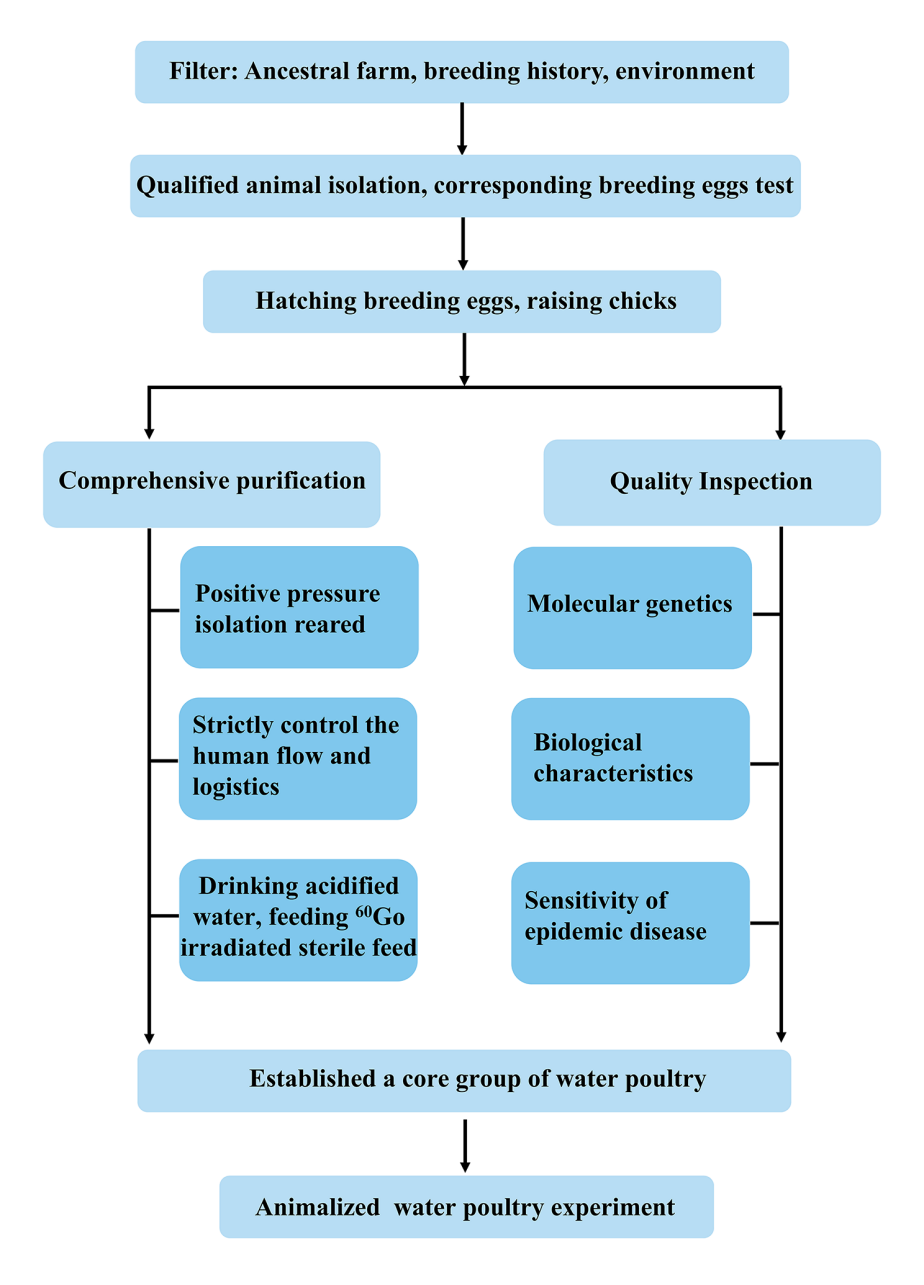
Cultivation flow chart of SPF ducks

SPF experimental ducks in this study represent the current breeding ducks in China and were maintained in an isolation environment at the National Poultry Laboratory Animal Resource Center (LARC). The cultivation of SPF ducks requires a series of strict processes (Fig. 1). In brief, all ducks were raised based on high-quality breeding eggs (SX are introduced from Shaoxing shelduck ancestor duck eggs of the Academy of Agricultural Sciences in Zhejiang Province; JD are introduced from the National Waterfowl Base Resource Library in Taizhou, Jiangsu; SM are introduced from the Shelduck Original Breeding Farm in Longyan City, Fujian), reared in positive pressure isolators in a barrier environment. The detection of pathogenic microorganisms was carried out in regular population surveys to eliminate positive individuals and ducks were confirmed to be free of duck hepatitis virus I, duck plague virus, duck circovirus, duck tembusu virus, goose parvovirus, avian leukosis viruses, avian reovirus, avian influenza virus (H5 subtype (Re8 strain), H7 subtype, H9 subtype), Newcastle disease virus, and avian adenovirus II. Using the wing vein method, we collected blood samples from 30 SPF ducks of similar body weight at 42 weeks old, including JD (5♀, 5♂), SX (5♀, 5♂), and SM (5♀, 5♂). Samples were immediately snap-frozen in liquid nitrogen for further extraction of genomic DNA. Table S1 shows the phenotypic descriptions of three breeds.

### Sequencing data processing

Genomic DNA was extracted from blood samples and paired-end libraries were constructed (insert size of 300–400 bp) using the Illumina NovaSeq 6000 platform (San Diego, CA, USA). Quality control was used to filter the reads to remove adapters and low-quality reads. The filtered raw reads were further processed to obtain high quality clean reads based on three strict filtering criteria: (1) removing reads with ≥ 10% unidentified nucleotides; (2) removing reads with > 50% bases having phred quality scores of ≤ 20; and (3) removing reads aligned to the barcode adapter. The clean reads were mapped to the *Anas platyrhynchos* reference genome obtained from the NCBI (https://www.ncbi.nlm.nih.gov/assembly/GCF_015476345.1) using the BWA software, with the following parameters: mem -t 4 -k 32 -M [31]. Duplicates were removed using SAMtools software [32]. If multiple read pairs had identical external coordinates, only the pair with the highest mapping quality were retained.

### Detection of CNVs and CNVRs

We firstly used CNVnator software to detect the CNVs in each duck genome sample [33]. Quality control was performed on the raw CNVs of each sample. The screening criteria included a *p*-value < 0.01 (e-val1 calculated using t-test statistics), size > 1 kb. CNV_type was judged based on the read depth (RD) value (deletion: RD < 0.7; duplication: RD > 1.3). Then, CNVcaller was used to detect population-level CNVRs [34]. To obtain high-confidence CNVs and CNVRs, we performed the following quality control procedures: (1) The CNVs of 10 samples from each group were fused using the “Merge” command of BEDTools [35]. (2) When overlapping sequences were at least > 1 bp along their genomic coordinates, we used the “intersect” command in BEDTools to merge multiple adjacent CNVs between individuals within a population into one CNVR and discarded each population or CNVRs that contained only one CNV in the metapopulation. We defined CNVRs containing only deletions as deleted CNVRs, duplicated CNVRs as duplicated CNVRs, and CNVRs containing both deletions and duplicates as complex CNVRs.

### Functional enrichment analysis of CNVR-harboring genes

CNVR-harboring genes were searched in the *Anas platyrhynchos* reference genome, and completely and partially (≥ 50%) overlapping genes were retained for subsequent analysis. Functional enrichment analysis using gene ontology (GO) and Kyoto Encyclopedia of Genes and Genomes (KEGG) [36], performed using the online tool DAVID (https://david.ncifcrf.gov/). A false discovery rate (FDR) < 0.05 was considered to indicate significant enrichment of candidate genes.

### Population genetics of CNVRs

Levels of genetic differentiation among populations were evaluated by using the Fixation index (*F*_ST_) method [37], using the -weir-fst-pop option in VCFtools [38]. Functional enrichment analysis was performed on the top 5% of CNVR loci showing extremely high *F*_ST_ values and tested whether these “outlier” loci were associated with important traits in ducks.

## Results

### Sequencing and CNV detection

**Fig. 2:**
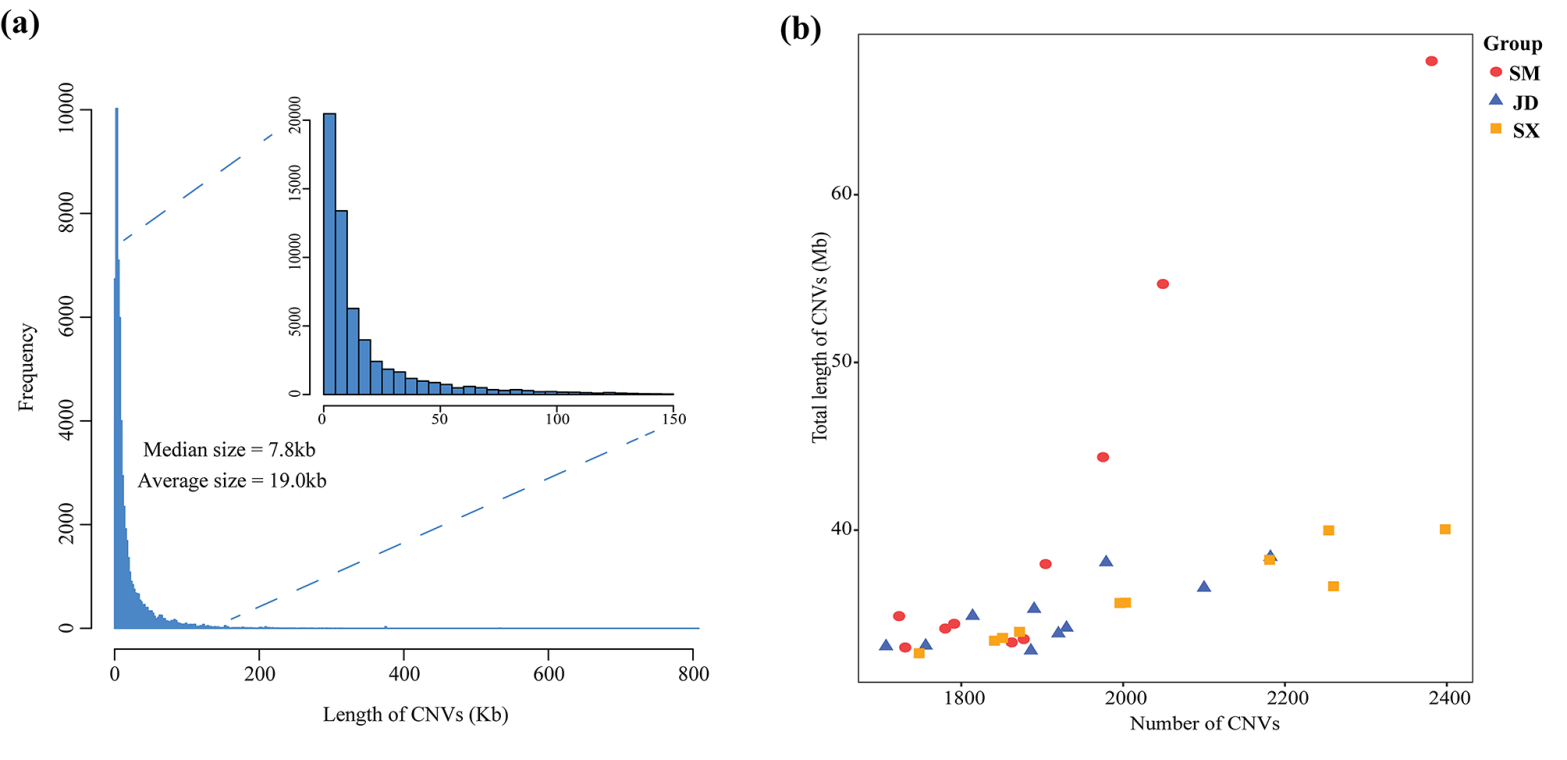
Genome-wide characterization of CNVs in the duck genome. (**a**) A histogram of the distribution of CNV length. (**b**) Total length and the total amount of CNVs identified in each sample

**Fig. 3:**
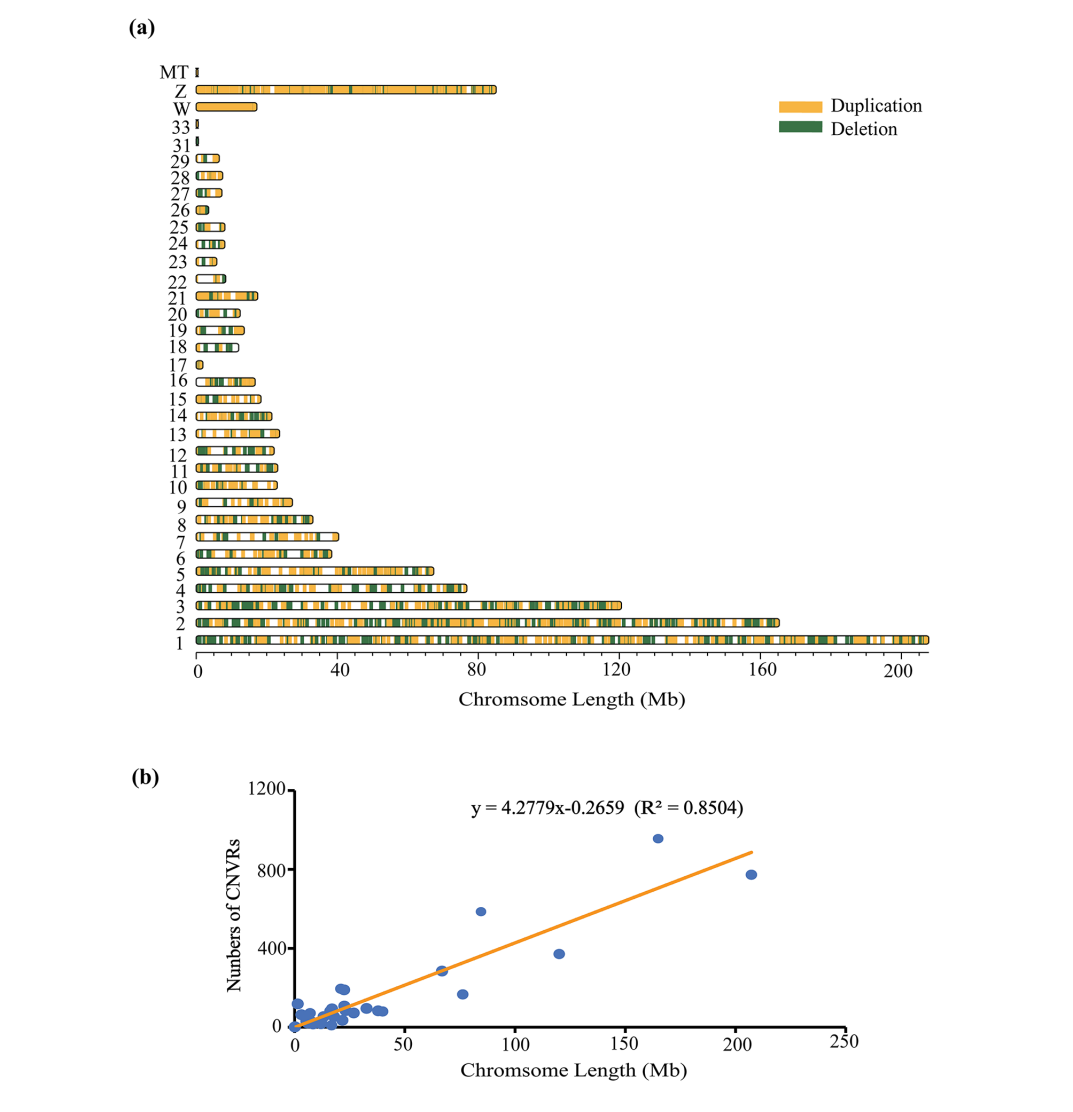
Genomic landscape of CNVRs. (**a**) A map of CNVRs in the duck genome; Two types of CNVR were identified, including duplication (yellow) and deletion (green). (**b**) Correlation between CNVR counts and chromosome length

Using Illumina paired-end sequencing technology, we obtained high-quality next generation sequencing data for 30 experimental ducks (Additional file 2: Table S2). The mapped read depth ranged from 19.65× to 28.33×, with an average depth of 22.65× per sample, indicating that these data were sufficient for further analysis (Additional file 3: Table S3). We detected a total of 73,012 CNVs, including 26,432 “duplication” events and 46,580 “deletion” events. The sizes of all the CNVs showed an L-shaped distribution (median size = 7.8 kb, average size = 19.0 kb) (Fig. 2a and Additional file 4: Table S4). At the individual level, we found an average of 1956 CNVs per duck genome, ranging from 1707 to 2398 (Fig. 2b and Additional file 5: Table S5). By merging overlapping CNVs, a total of 4,810 CNVRs were obtained, covering 4.2% of the duck genome (Additional file 6: Table S6). Among them, 2,263; 2,127; and 2,128 CNVRs were obtained in the SM, JD and SX ducks, respectively (Additional file 7: Fig. S1). There was a significant positive linear relationship between the number of CNVRs and the corresponding autosomal size (R^2^ = 0.85, Fig. 3).

### Functional annotation of the identified CNVRs

**Fig. 4:**
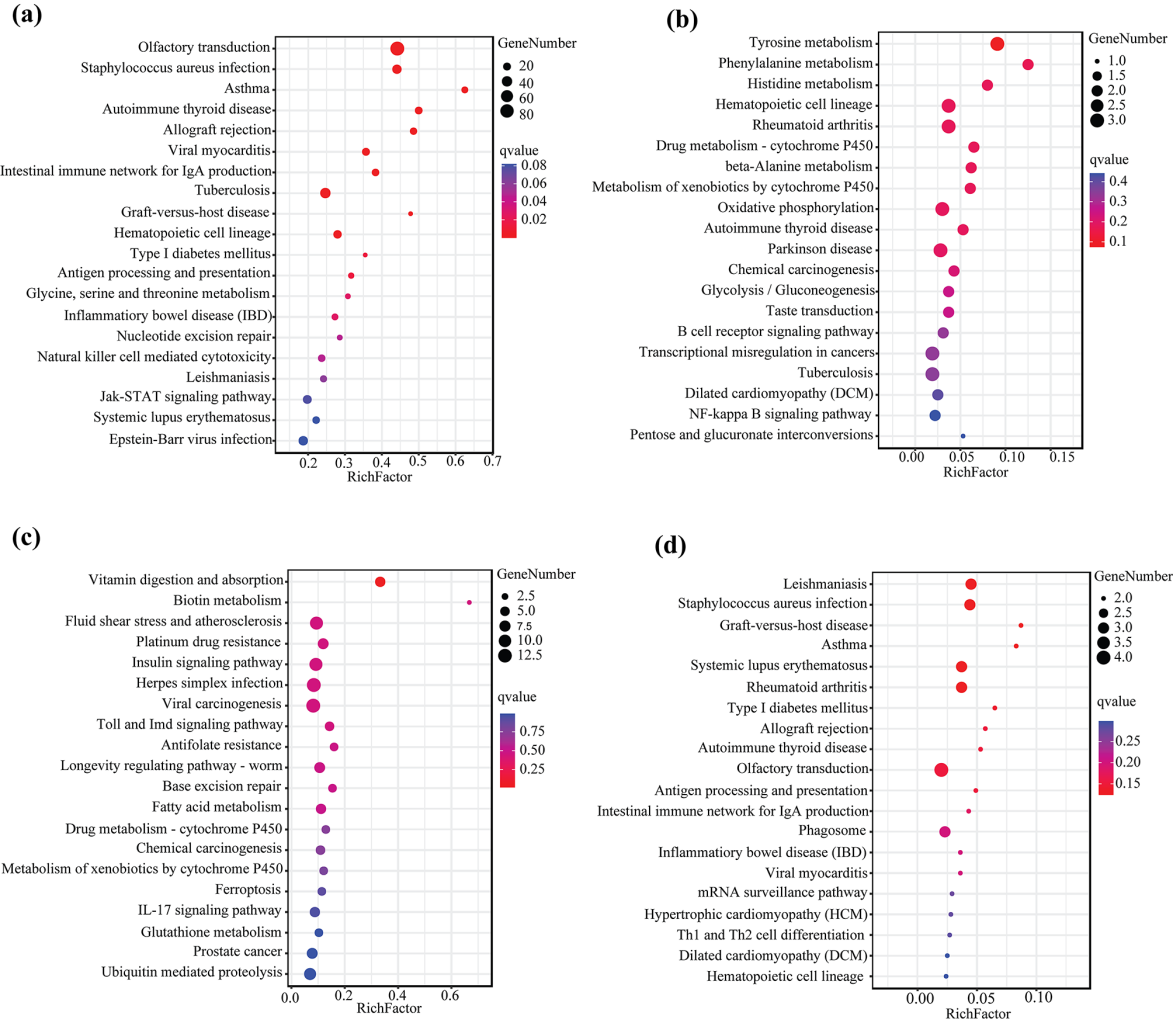
KEGG pathway enrichment analysis (www.kegg.jp/kegg/kegg1.html). (**a**) Top 20 enriched signaling pathways of CNVR-harbored genes shared among the three duck populations. (**b**) Top 20 enriched signaling pathways for JD-, (**c**) SM-, and (**d**) SX-specific CNVR-harbored genes

From the genome annotation, there were 750, 97, and 92 CNVR-harboring genes detected only in SM, JD, and SX, respectively, while 2675 CNVR-harboring genes were detected in all three populations (Additional file 8: Fig. S2). The functional enrichment analysis showed that 31 GO terms were enriched in the CNVRharboring genes shared by the three populations, comprising 6 biological processes, 9 cellular components, and 16 molecular functions. These GO terms were mainly related to olfactory receptor activity (GO:0004984) and signaling receptor activity (GO:0004888, GO:0060089, GO:0038023, and GO:0004930) (Additional file 9: Table S7). The KEGG pathway analysis identified 16 significantly enriched pathways, including olfactory transduction (ko04740) and the immune system (ko05320, ko04612, and ko04650) (Fig. 4 and Additional file 10: Table S8). Furthermore, we performed functional enrichment analysis of specific CNVR-harboring genes in the three duck populations. In particular, the CNVR-harboring genes specifically distributed in SM were mainly involved in oxygen transporter activity (GO:0005344) and oxygen binding (GO:0019825).

### Population genetics of CNVRs

**Fig. 5:**
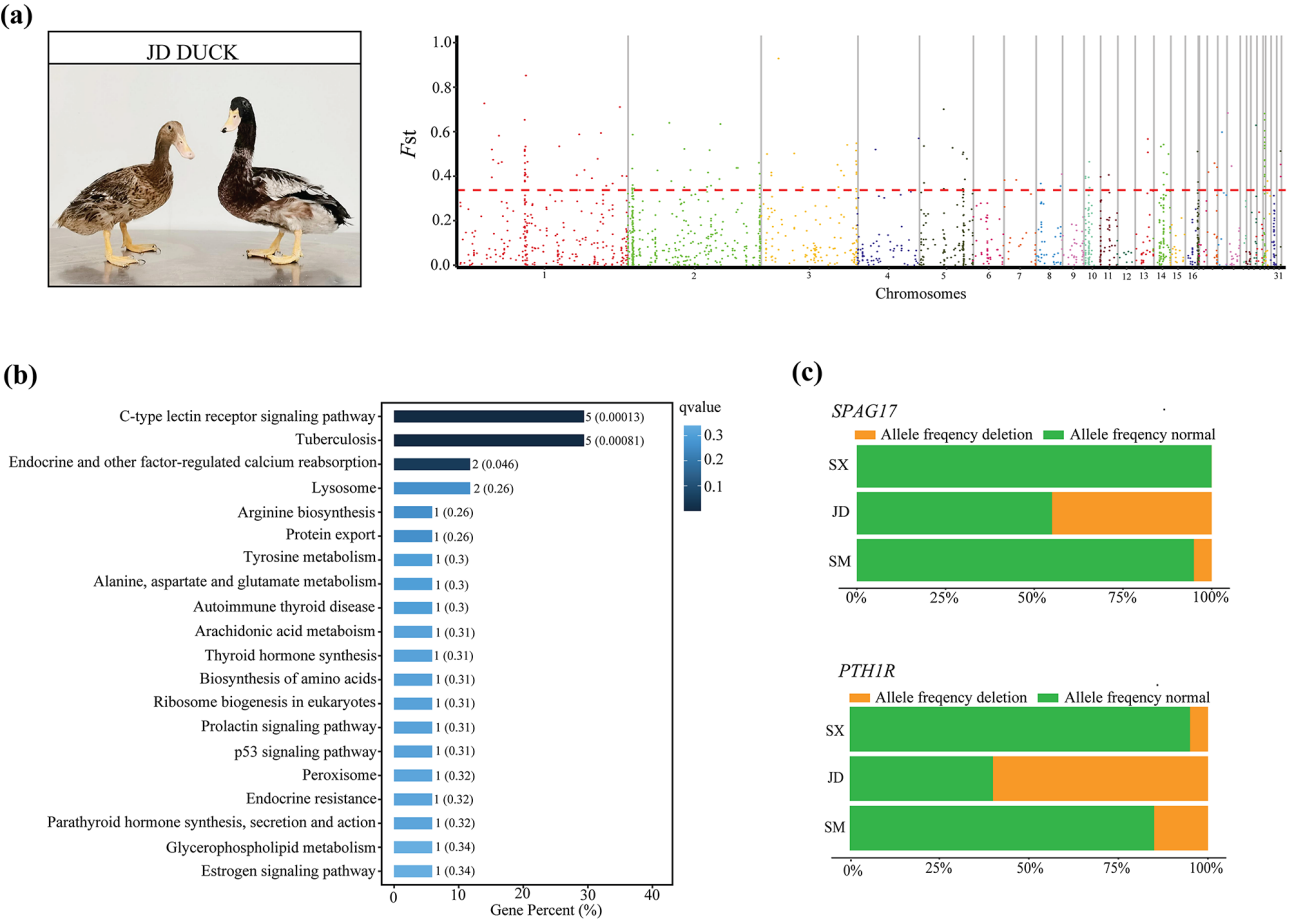
Comparative genomic analysis for JD vs. SX and SM using population fixation index (*F*_ST_). (**a**) Manhattan plot of genome-wide *F*_ST_ on each CNVR locus between for JD vs. SX and SM. (**b**) Top twenty enriched KEGG pathways for the genes overlapped with highly differentiated CNVRs for JD vs. SX and SM (www.kegg.jp/kegg/kegg1.html). (**c**) Allele frequencies of *SPAG17* and *PTH1R*

Through estimating the genome-wide *F*_ST_ to detect the CNVRs that were genetically differentiated in each duck population, we were able to perform the following comparisons: CNVRs from JD compared with those from SX and SM, CNVRs from SM compared with those from JD and SX, and CNVRs from SX compared with those from JD and SM. Based on the top 5% of the *F*_ST_ distribution, 159 outlier loci that overlapped with 56 genes were considered highly divergent in JD (Fig. 5a and Additional file 11: Table S9). The functional analysis identified three significantly enriched pathways, including the C-type lectin receptor signaling pathway (ko04625), tuberculosis (ko05152) and endocrine and other factor-regulated calcium reabsorption (ko04961) (Fig. 5b and Additional file 12: Table S10). Among all CNVRs analyzed, we identified a 2,000 bp deletion (chromosome 1: 83,517,601–83,519,600 bp) in *SPAG17* (encoding sperm-associated antigen 17) and a 1,600 bp deletion (chromosome 2: 163,037,601–163,039,200 bp) in *PTH1R* (encoding parathyroid hormone 1 receptor). The frequencies of their deletion were high in JD, but lower in the other populations (Fig. 5c).

**Fig. 6:**
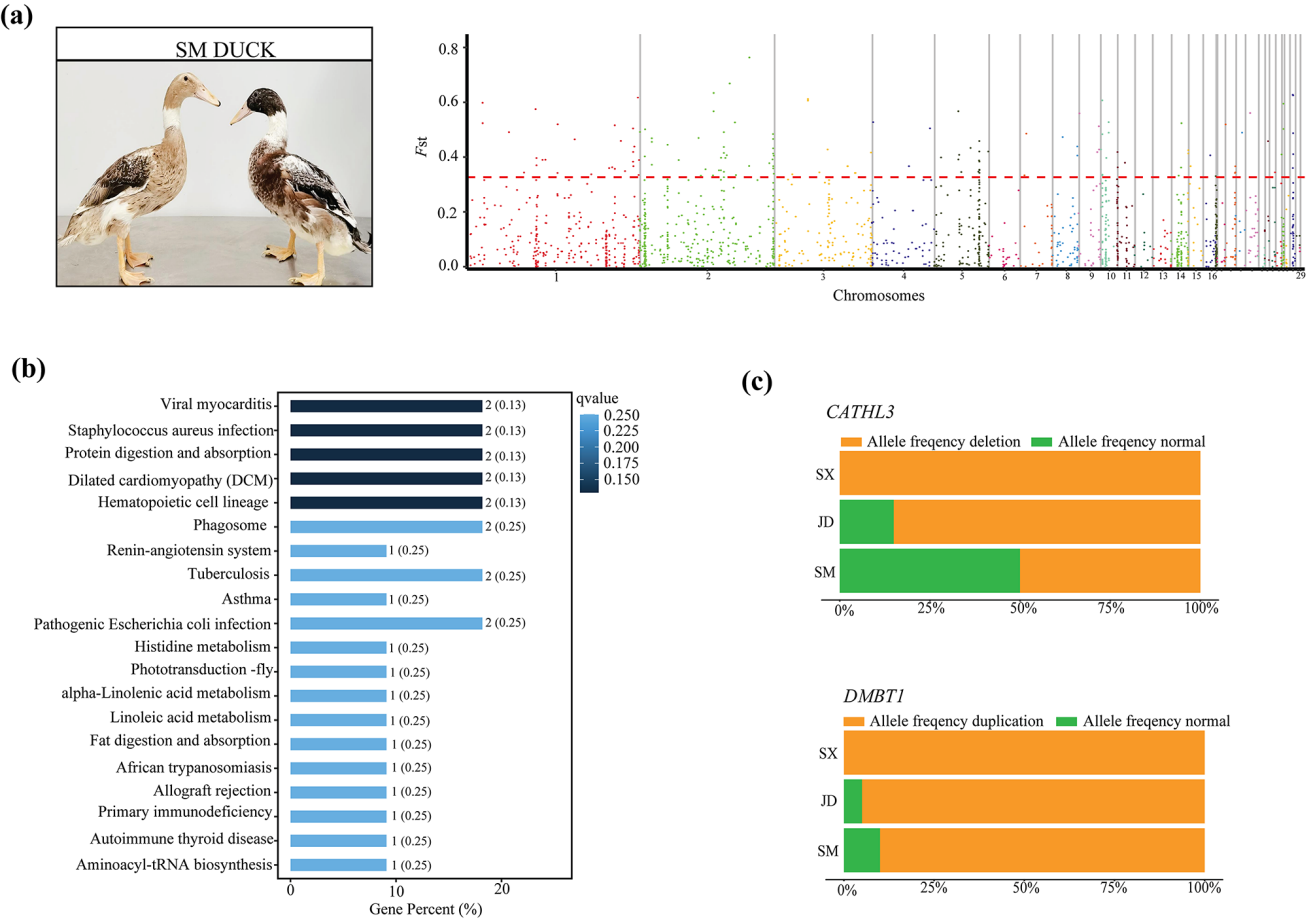
Comparative genomic analysis for SM vs. JD and SX using population fixation index (*F*_ST_). (**a**) Manhattan plot of genome-wide *F*_ST_ on each CNVR locus between for SM vs. JD and SX. (**b**) Top twenty enriched KEGG pathways for the genes overlapped with highly differentiated CNVRs for SM vs. JD and SX (www.kegg.jp/kegg/kegg1.html). (**c**) Allele frequencies of *CATHL3* and *DMBT1*

Based on the top 5% of the empirical *F*_ST_ distribution, 159 outlier loci that overlapped with 51 genes were considered highly divergent in SM (Fig. 6a and Additional file 11: Table S9). KEGG pathway analysis showed that the enriched differentiated CNVR genes were mainly associated with viral myocarditis (ko05416), *Staphylococcus aureus* infection (ko05150) and protein digestion and absorption (ko04974) (Fig. 6b and Additional file 12: Table S10). The genome-wide distribution of *F*_ST_ showed that the most significantly variation was a 3,200 bp deletion (chromosome 2: 163,312,001–163,315,200 bp) that overlapped with the *CATHL3* gene (encoding cathelicidin 3) and a 3,600 bp duplication (chromosome 5: 55,372,001–55,375,600 bp) overlapping with the *DMBT1* gene (encoding deleted in malignant brain tumors 1). The frequencies of these CNVRs in SM were lower relative to that those in the other populations (Fig. 6c).

**Fig. 7:**
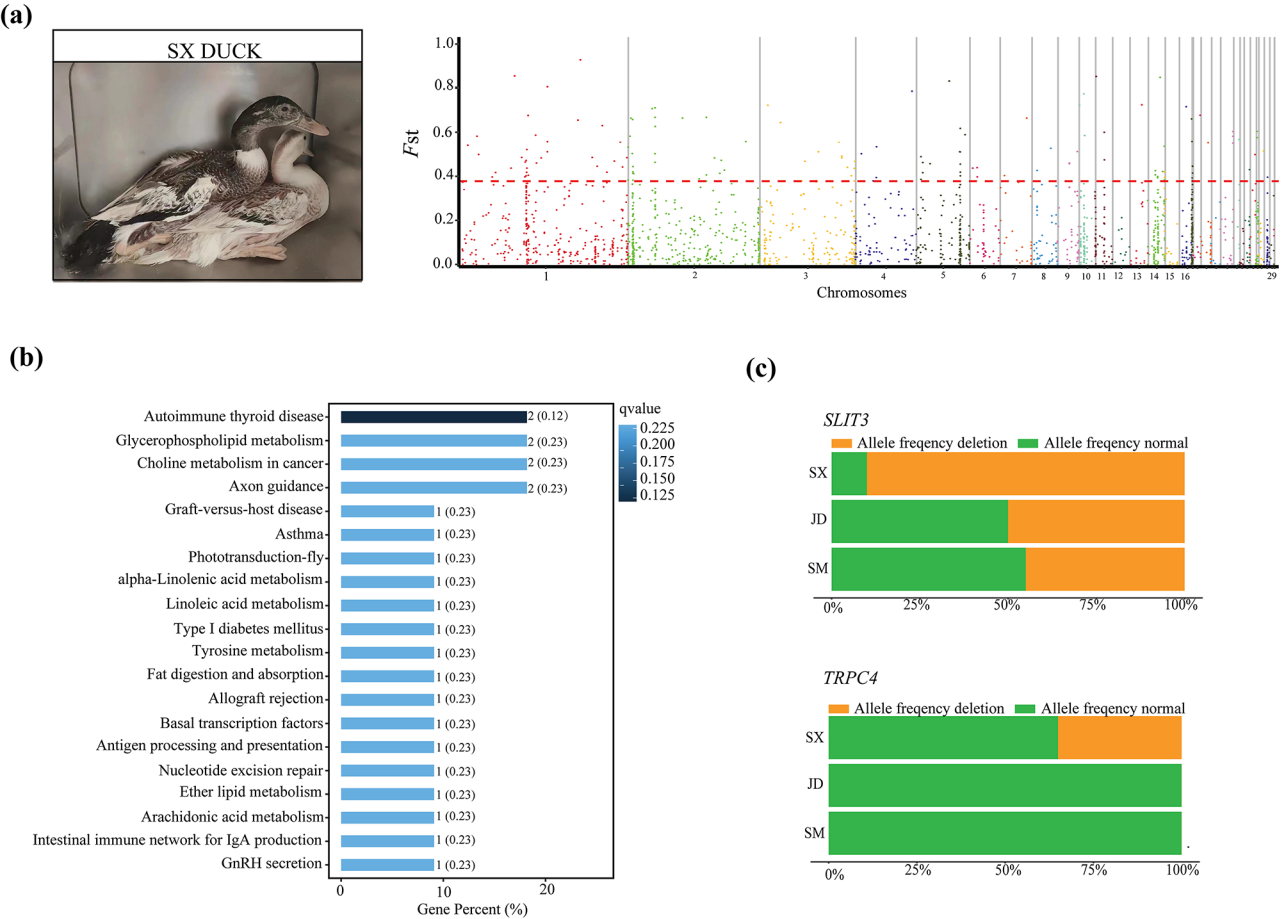
Comparative genomic analysis for SX vs. JD and SM using population fixation index (*F*_ST_). (**a**) Manhattan plot of genome-wide *F*_ST_ on each CNVR locus between for SX vs. JD and SM. (**b**) Top twenty enriched KEGG pathways for the genes overlapped with highly differentiated CNVRs for SX vs. JD and SM (www.kegg.jp/kegg/kegg1.html). (**c**) Allele frequencies of *TRPC4* and *SLIT3*

In the selective sweep analyses, we identified 161 outlier loci overlapping with 65 genes that were considered highly divergent in SX (Fig. 7a and Additional file 11: Table S9). Functional analysis revealed that genes overlapping with differentiated CNVRs were enriched in autoimmune thyroid disease (ko05320) and glycerophospholipid metabolism (ko00564) (Fig. 7b and Additional file 12: Table S10). Additionally, we identified the strongest selection signal as a 2,000 bp deletion (chromosome 14: 15,796,801–15,798,800 bp) containing the *SLIT3* gene (encoding slit guidance ligand 3) and a 2,400 bp deletion (chromosome 1: 181,252,801–181,255,200 bp) containing the *TRPC4* gene (encoding transient receptor potential channel 4), the deletion frequencies of which were high in SX, but much lower in the other populations (Fig. 7c).

## Discussion

Understanding the genetic basis of phenotypic differences is a major theme in animal science. CNVs, as important sources of genetic diversity, have attracted widespread attention in the last decade because of their dramatic phenotypic consequences [39, 40]. Studies have shown that the average size of avian genomes and the range of variation in genome size are the smallest among all vertebrate groups (http://www.genomesize.com) and that the overall karyotype structure is highly conserved [41]. In addition, the number of CNVs is also lower in avian genomes compared with that in mammalian genomes [42]. Therefore, avian genomes are particularly suitable to analyze CNVs because of their unique combination of features [41]. However, research related to avian CNVs (especially in ducks) is scarce. Herein, we detected the CNVs of three experimental duck species in China based on the WGRS technique, which identified 2,263; 2,127; and 2,128 CNVRs for SM, JD, and SX ducks respectively by merging 73,012 CNVs from all duck samples. Compared with previous studies based on 600 K SNP chip array chicken CNV profiling [43], we detected about twice as many CNVRs per duck population, which is consistent with comparative genomics studies of chickens and Peking ducks reported by a previous study [26]. Furthermore, we found that the CNVRs accounted for 4.2% of the duck reference genome, whereas they accounted for 5.12% in chickens [43], 6.2% in yaks (*Bos grunniens*) [44], and 10.8% in goats [45]. The different number of samples, different detection methods, and different reference genomes likely contributed to the observed inconsistencies in CNVR counts. Our findings further complemented the research base of duck CNVRs. Notably, this study is the first to focus on the genomic CNVR maps of different experimental duck populations.

Genes located in CNVR regions provide a resource to study the biological relationships between CNVRs and the genetic basis of phenotypic variation caused by their broad molecular functions. The GO enrichment analysis revealed that the detected CNVRs of the three populations shared genes that were mostly enriched in terms of olfactory receptor activity. The term olfactory receptor activity is the combination of an olfactory receptor and an odor, manifested by the transmission of a signal from one side of the membrane to the other in response to odor detection [46]. Odor is crucial to animal survival, because it contributes to the animal’s hedonic evaluation of food, thereby effectively assisting the animal in choosing food and its possible consumption [47]. Previous studies have also reported an association of olfactory transduction with feed efficiency in cattle and their production properties [48], and the remaining feed intake of pigs [49]. The experimental ducks used in this study were reared in a positive pressure isolator under a barrier environment, drinking acidified water, and were fed ^60^Co radiation sterile feed. This suggests that the high rate of olfactory receptor CNVR variability might help species adapt to specific environments more quickly by exerting in appetite regulation. The results of the KEGG signaling pathway analysis showed that some CNVR-harboring genes were enriched in signaling pathways related to the immune system, such as antigen processing and presentation, and autoimmune thyroid disease. As experimental ducks, they face specific living conditions. Strict purification treatment involves blocking environmental pollution and re-infection pathways, regularly monitoring the quantity of pathogenic microorganisms, and eliminating positive individuals to ensure their freedom from epidemic diseases. Previous studies on SPF/non-SPF animals in histology [50], physiology, biochemistry [51] and epidemic susceptibility [52] revealed that SPF animals have a high susceptibility to pathogens, but stable genetic properties, making them a valuable resource for disease and immune research. Furthermore, immune-related genes evolve at a rapid rate [53, 54]. Thus, modulation of the immune system during pathogen-free cultivation is predictable. Collectively, the enriched CNVR overlapping genes related to olfactory receptors and the immune system might help us to understand the common environmental adaptation mechanisms. Notably, the oxygen transporter activity and oxygen binding pathways were significantly and specifically enriched in SM. These pathways have been identified in animals such as Tibetan sheep [55] and Tibetan chickens [56] to explain their adaptation to hypoxia. This coincides with the actual situation of this duck population. The production area of SM in Longyan Reserve in Fujian Province is mostly composed of mountainous areas, suggesting that SM can survive well in an environment with a limited oxygen supply.

Selection signature analysis based on sequencing data can reveal genomic regions that have undergone artificial selection and environmental change during local adaptation and evolution [57]. To screen for selection regions and genes specific to each population, the *F*_ST_ values for one population of experimental ducks compared with those of the other populations. Herein, the CNVR harboring genes *SPAG17* and *PTH1R* showed significant differentiation in JD. *SPAG17* encodes a multifunctional cytoplasmic protein that not only affects reproduction, but also is used extensively in the analysis of body-measurement traits related to skeletal development [58]. *SPAG17* plays a crucial role in determining human body height. SNPs of *SPAG17* have been reported to be associated with height and idiopathic short stature in infants [59], children [60], and adults [61]. In the livestock industry, *SPAG17* expression is often used as growth trait data to guide the scientific raising and breeding of animals, as reported in goats [58] and cattle [62]. PTH1R also plays an important role in skeletal homeostasis. After PTH activation of PTH1R, it mediates catabolic and anabolic processes in bone. *PTHR1* gene mutation causes Jansen’s metaphyseal chondrodysplasia [63]. In another study, a 51 bp indel polymorphism in the *PTH1R* gene was associated with growth and carcass traits in chickens [64]. Therefore, the identification *SPAG17* and *PTH1R* further deepened our understanding of the genetic mechanisms underlying growth traits in JD.

Although related issues have been extensively studied through SNPs, there have been few reports on CNVR-based selection signals for adaptation to disease resistance in humans and animals [65, 66]. In the present study, we highlighted genes (*CATHL3* and *DMBT*) that overlapped highly differentiated CNVRs between SM and other duck populations. CATHL3 is a small cationic antimicrobial peptide with effective activity against a wide range of pathogens, including bacteria, viruses, and fungi [67]. Previous studies have confirmed that *CATHL3* is a potential candidate gene related to disease resistance studies in humans [68] and Gir cattle (*Bos indicus*) [69]. Similarly, DMBT1, a member of the scavenger receptor cysteine-rich super family, is considered to play a role in tumorigenesis and pathogen defense [70]. A *DMBT1*-harbored SNP selection signal provides evidence of a bovine tuberculosis (bTB) susceptibility gene in cattle breeds [65]. SM inhabits a mountainous area that has been relatively closed to transportation for a long time, acting as a natural barrier to some extent. In addition, SM exists mainly in free-ranging populations with less vaccination during the breeding process. Therefore, we speculated that CNVR-harbored *CATHL3* and *DMBT1* have undergone natural selection by mountain ecology in SM, with possible importance in disease resistance. Further studies are warranted to characterize the causal relationship between these genes and disease resistance in SM.

Temperature stress (high or low temperatures) is one of the most serious environmental challenges facing poultry worldwide, with negative effects on duck health, welfare and productivity. Organisms can assess changes in environmental temperature to produce certain physiological and behavioral responses that benefit survival. The activation of certain ion channels of the transient receptor potential (TRP) family by changes in ambient temperature, as well as the identification of their heterogeneous expression patterns and heterogeneous temperature sensitivity, have triggered the interest of researchers to evaluate these proteins as candidate endogenous thermosensors [71]. TRPC4 has been identified as a promising molecular target for body temperature management. Loss-of-function studies of TRPC4 demonstrated its function in GABAergic warm sensitive neurons, resulting in extra deficits in basal temperature setting, warm defense, and fever responses [72]. Recent studies have reported that TRPC4 is associated with thermoregulation [73] in cattle and cold adaptation [74] in Arctic dogs. In addition, secretion of the macrophage cytokine SLIT3 by adipose tissue macrophages enhances cold adaptation via stimulating sympathetic nerves and thermogenesis in mice (*Mus musculus*) [75]. In this study, the highly differentiated CNVRs between SX and the other duck populations overlapped with *TRPC4* and *SLIT3*. We hypothesized that *TRPC4* and *SLIT3* might be involved in thermoregulation in SX. Furthermore, functional analysis revealed that the autoimmune thyroid disease pathway was the most significantly enriched among all pathways for SX-differentiated CNVR genes. Studies have shown that thyroid disorders potentially interfere with the normal regulation of body temperature in humans [76]. Thyroid hormone synthesis is increased in birds and mammals in cold environments. The size and activity of the thyroid also increase especially at low temperatures [74, 77]. It has been reported that the expression of the *TPO* gene (encoding thyroid peroxidase, a key factor of the autoimmune thyroid disease pathway) is up-regulated in Bashang long-tail chicken (BS) and Rhode Island red chickens (RIR) in cold environments [78]. Therefore, we speculated that this pathway might also be related to the thermoregulation of SX. However, more functional experiments are necessary to fully reveal their biological functions.

## Conclusion

In the present study, the first resequencing based CNV map of experimental SPF ducks was developed. Functional enrichment analysis of the identified genes in shared CNVRs revealed several underlying biological processes responsible for olfactory receptors and the immune system of experimental ducks. Selective sweep analysis showed that growth (*SPAG17* and *PTH1R*), disease resistance (*CATHL3* and *DMBT1*), and thermoregulation (*TRPC4* and *SLIT3*) candidate gene were identified in strongly selected signatures specific to JD, SM, and SX, respectively. Although these phenotype-associated genes need to be further validated by biological experiments, our findings provide valuable information to identify the molecular basis of important phenotypic variations in experimental ducks.

### Electronic supplementary material

Below is the link to the electronic supplementary material.

Additional file 1: Table S1. Phenotypic description of three SPF ducks

Additional file 2: Table S2. Number of reads in the quality control

Additional file 3: Table S3. Overview of sample mapping statistics and depth coverage

Additional file 4: Table S4. List of CNVs identified in each individual

Additional file 5: Table S5. Total length and the total amount of CNVs identified in each sample

Additional file 6: Table S6. List of CNVRs identified in each individual

Additional file 7: Figure S1. Summary of CNVRs identified in the three duck populations

Additional file 8: Figure S2. Venn diagram of CNVR numbers identified in three duck populations

Additional file 9: Table S7. Gene ontology functional enrichment of CNVR-harbored genes

Additional file 10: Table S8. Kyoto Encyclopedia of Genes and Genomes (KEGG) functional enrichment of CNVR-harbored genes

Additional file 11: Table S9. List of highly differentiated CNVRs and annotated genes

Additional file 12: Table S10. KEGG analysis of CNVR-harbored that are genes differentially expressed in three duck populations

## Data Availability

The raw sequence data files from this study have been deposited in SRA and BioProject ID is PRJNA896757 (https://www.ncbi.nlm.nih.gov/sra/PRJNA896757).

## Abbreviations

CNV: Copy number variation
CNVRs: Copy number variation regions
JD: Jinding ducks
SX: Shaoxing ducks
SM: Fujian Shanma ducks
SPF: Specific pathogen-free
SNP: Single nucleotide polymorphism
WGRS: Whole-genome resequencing
aCGH: Array comparative genomic hybridization
RD: Read depth
*F*_ST_: Fixation index
GO: Gene ontology
KEGG: Kyoto encyclopedia of genes and genomes
*SPAG17*: Sperm-associated antigen 17
*PTH1R*: Parathyroid hormone 1 receptor
*CATHL3*: Cathelicidin 3
*DMBT1*: Deleted in malignant brain tumors 1
*SLIT3*: Slit guidance ligand 3
*TRPC4*: Transient receptor potential channel 4
