# Supplementary figures and images for: Identification of the genetic characteristics of copy number variations in experimental specific pathogen-free ducks using whole-genome resequencing
Source: BMC Genomics. 2024 Jan 2;25:17. doi: 10.1186/s12864-023-09928-8 (PMC10759622; doi:10.1186/s12864-023-09928-8)

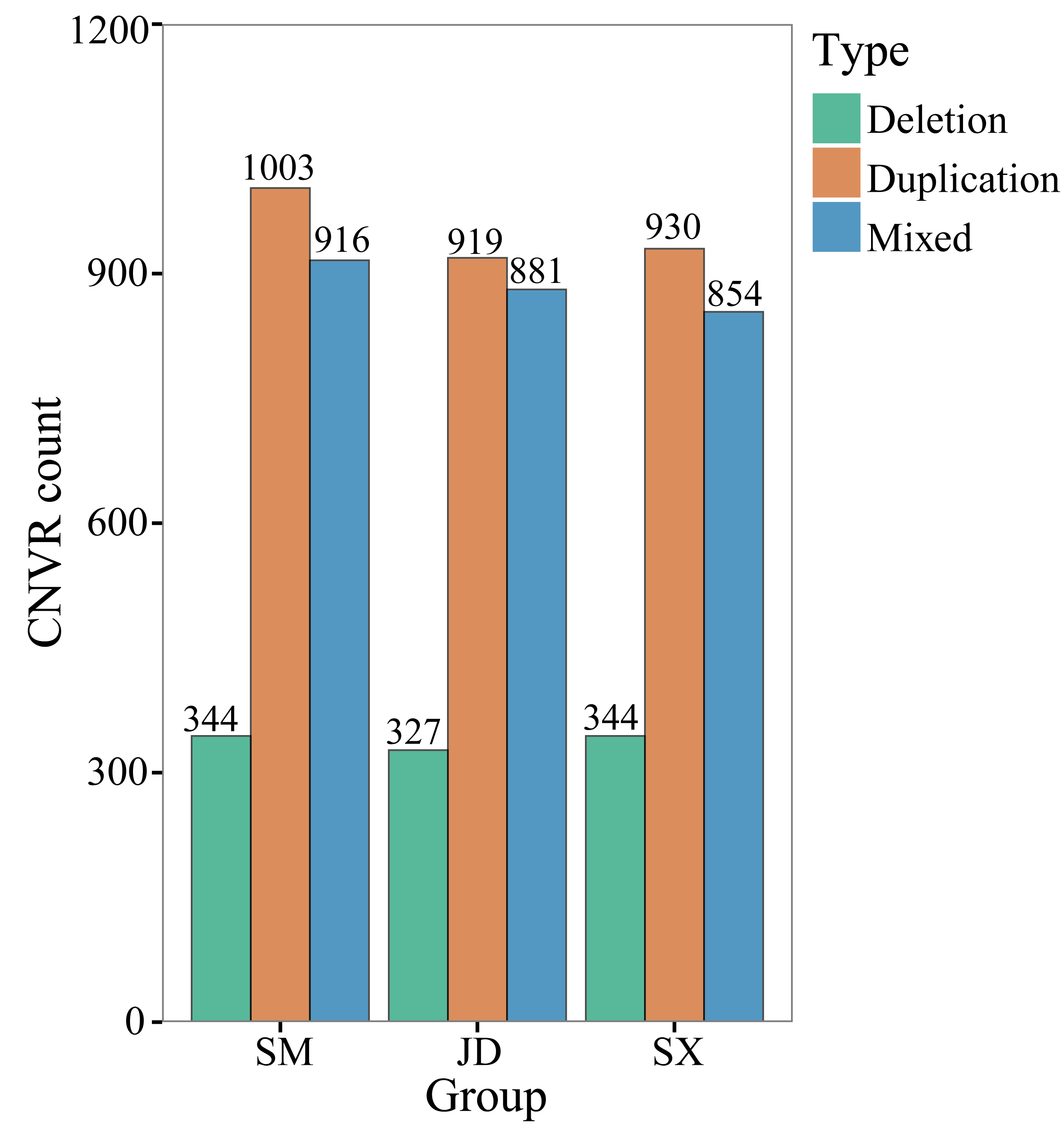

Supplement: Supplementary file 7 — Additional file 7: Figure S1. Summary of CNVRs identified in the three duck populations [file 12864_2023_9928_MOESM7_ESM.png]

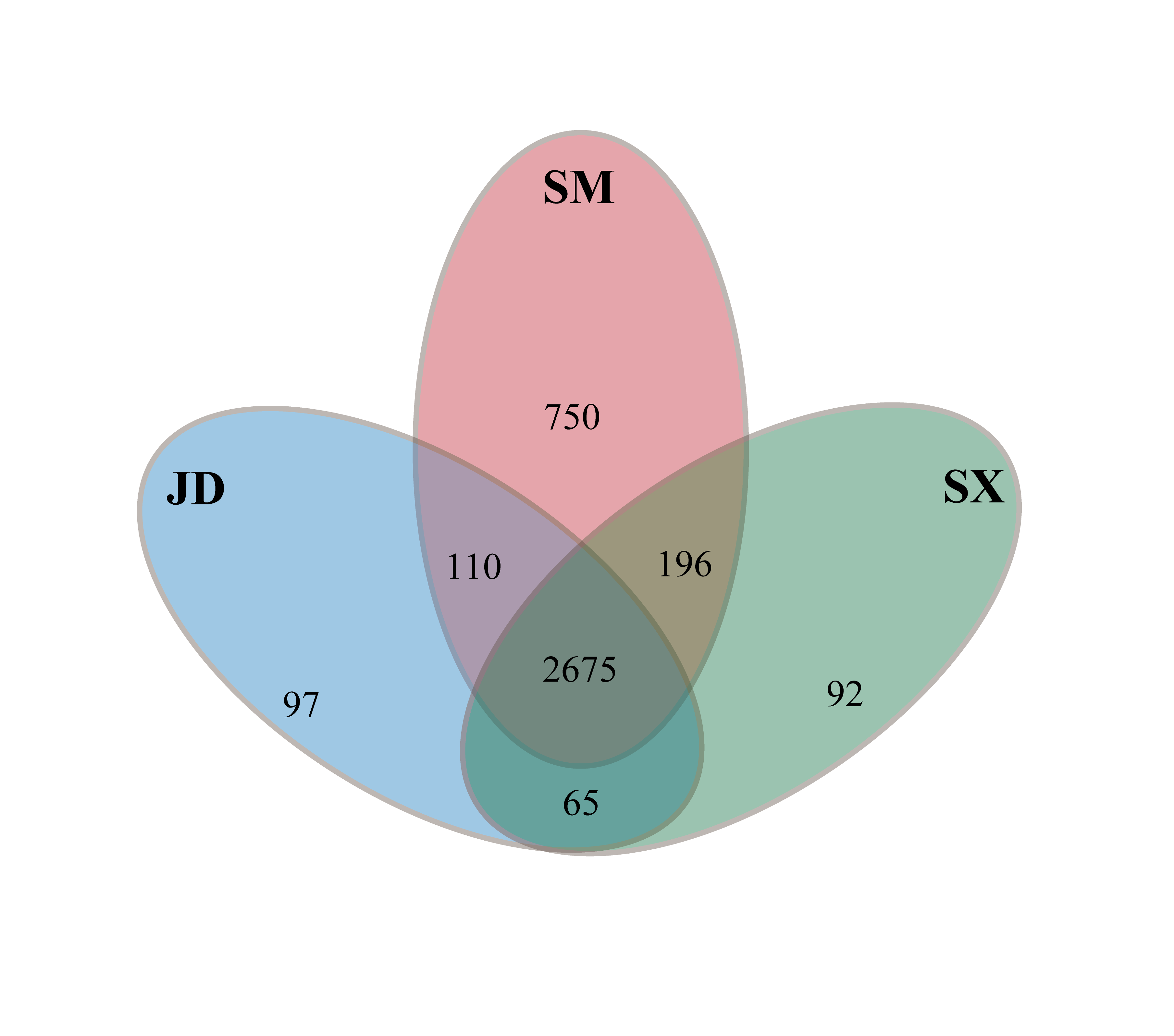

Supplement: Supplementary file 8 — Additional file 8: Figure S2. Venn diagram of CNVR numbers identified in three duck populations [file 12864_2023_9928_MOESM8_ESM.png]
